# Experimental Type 2 Diabetes Induces Enzymatic Changes in Isolated Rat Enterocytes

**DOI:** 10.1155/EDR.2003.119

**Published:** 2003

**Authors:** Isabel M. Martínez, Inmaculada Morales, Guadalupe García-Pino, José E. Campillo, María A. Tormo

**Affiliations:** 1 Departamento de Fisiología Facultad de Medicina Universidad de Extremadura Avda. de Elvas s/n Badajoz E-0607 Spain

## Abstract

Diabetes in humans and in experimental animals produces changes in the function and structure of the small intestine. The authors determined the activity of intestinal disaccharidases (maltase and sucrase) and of 6-phosphofructo-1-kinase (PFK-1) in enterocytes isolated from the small intestine of male Wistar rats (2.5 to 3 months old) with experimental nonobese type 2 diabetes, induced by streptozotocin (STZ) injection on the day of birth (n0-STZ) or on the 5th day of life (n5-STZ), with different degrees of hyperglycemia and insulinemia (n0-STZ and n5-STZ models). The glycemia (mmol/L) of the diabetic rats (n0-STZ: 8.77 ± 0.47; n5-STZ: 20.83 ± 0.63) was higher (*P* < .01) than that of the nondiabetic (ND) rats (5.99 ± 0.63); on the contrary, the insulinemia (ng/mL) was significantly lower in both n0-STZ (1.74 ± 0.53; *P* < .05) and n5-STZ (1.12 ± 0.44; *P* < .01) diabetic rats than in normal rats (3.77 ± 0.22). The sucrase and maltase activities (U/g protein) in diabetic rats (n0-STZ: 89 ± 9 and 266 ± 12; n5-STZ: 142 ± 23 and 451 ± 57) were significantly higher than those in the ND group (66 ± 5 and 228 ± 22). The PFK-1 activities (mU/mg protein) in the diabetic models (n0-STZ: 14.89 ± 1.51; n5-STZ: 13.35 ± 3.12) were significantly lower (*P* < .05) than in ND rats (20.54 ± 2.83). The data demonstrated enzymatic alterations in enterocytes isolated fromthe small intestine of n0-STZ rats that are greater (*P* < .05) than in the more hyperglycemic and hypoinsulinemic n5-STZ animals. The results also show that nonobese type 2–like diabetes in the rat produces modifications that favor an increase in glucose absorption rates.


*Experimental Diab. Res.*, 4:119–123, 2003
Copyright © Taylor and Francis Inc.
ISSN: 1543-8600 print / 1543-8619 online
DOI: 10.1080/15438600390214455

Taylor & Francis
Taylor & Francis Group

### Experimental Type 2 Diabetes Induces Enzymatic Changes in Isolated Rat Enterocytes

**Isabel M. Martínez, Inmaculada Morales, Guadalupe García-Pino, José E. Campillo, and María A. Tormo**

*Departamento de Fisiología, Facultad de Medicina, Universidad de Extremadura, Badajoz, Spain*

**Diabetes in humans and in experimental animals produces changes in the function and structure of the small intestine. The authors determined the activity of intestinal disaccharidases (maltase and sucrase) and of 6-phosphofructo-1-kinase (PFK-1) in enterocytes isolated from the small intestine of male Wistar rats (2.5 to 3 months old) with experimental nonobese type 2 diabetes, induced by streptozotocin (STZ) injection on the day of birth (n0-STZ) or on the 5th day of life (n5-STZ), with different degrees of hyperglycemia and insulinemia (n0-STZ and n5-STZ models). The glycemia (mmol/L) of the diabetic rats (n0-STZ: $8.77 \pm 0.47$; n5-STZ: $20.83 \pm 0.63$) was higher ($P < .01$) than that of the nondiabetic (ND) rats ($5.99 \pm 0.63$); on the contrary, the insulinemia (ng/mL) was significantly lower in both n0-STZ ($1.74 \pm 0.53$; $P < .05$) and n5-STZ ($1.12 \pm 0.44$; $P < .01$) diabetic rats than in normal rats ($3.77 \pm 0.22$). The sucrase and maltase activities (U/g protein) in diabetic rats (n0-STZ: $89 \pm 9$ and $266 \pm 12$; n5-STZ: $142 \pm 23$ and $451 \pm 57$) were significantly higher than those in the ND group ($66 \pm 5$ and $228 \pm 22$). The PFK-1 activities (mU/mg protein) in the diabetic models (n0-STZ: $14.89 \pm 1.51$; n5-STZ: $13.35 \pm 3.12$) were significantly lower ($P < .05$) than in ND rats ($20.54 \pm 2.83$). The data demonstrated enzymatic alterations in enterocytes isolated from the small intestine of n0-STZ rats that are greater ($P < .05$) than in the more hyperglycemic and hypoinsulinemic n5-STZ animals. The results also show that nonobese type 2–like diabetes in the rat produces modifications that favor an increase in glucose absorption rates.**

**Keywords** Enterocyte; Maltase; Nonobese Type 2–like Diabetes; 6-Phosphofructo-1-kinase; Sucrase

In humans and in experimental animals, diabetes produces changes in the function and structure of the small intestine, including increased glucose transport [1, 2], increased specific and total disaccharidase activity [3–6], and changes in 6-phosphofructo-1-kinase activity [7, 8]. All these alterations can contribute to the appearance of postprandial hyperglycemic peaks, and consequently to the development of the chronic complications of diabetes.

Most studies demonstrating the influence of diabetes on the intestine have been performed in models of diabetes induced in adult experimental animals by the administration of alloxan or streptozotocin (STZ). This leads to a diabetes similar to type 1 in man, with a complete absence of plasma insulin and extreme hyperglycemia. There are few data, however, from other diabetes models characterized by glycemia levels not as high as those in type 1 diabetes and by a wide range of plasma insulin levels. The exact mechanisms of these enzymatic alterations in diabetes are unknown, but some studies on animals with experimental type 1 diabetes have suggested that the hyperglycemia [9] might in part be responsible for an increased disaccharidase activity, which is reduced after insulin treatment [2].

Given this situation, our purpose was to study the activity of the intestinal disaccharidases and of 6-phosphofructo-1-kinase, in enterocytes isolated from the small intestine of 2 rat models of nonobese type 2 diabetes with different degrees of hyperglycemia and insulinemia.

Received 8 December 2002; accepted 27 March 2003.

This work was supported by grants from the Spanish Comision Interministerial de Ciencia y Tecnología (CICYT) (no. ALI98-0706) and from the Junta de Extremadura–Consejería de Educación y Fondo Social Europeo (no. IPR00C037).

Address correspondence to M. A. Tormo, Departamento de Fisiología, Facultad de Medicina, Universidad de Extremadura, Avda. de Elvas s/n, E-06071 Badajoz, Spain. E-mail: matormo@unex.es

### MATERIALS AND METHODS

### Experimental Animals

We used male Wistar rats, fed a standard commercial diet (maintenance diet Letica, Panlab S.L., Barcelona, Spain; 61.41% *w/w* carbohydrate, 3.96% fiber, 15.06% protein, and 2.66% fat). The rats had free access to water and food. They were housed in the animalarium of the University of Extremadura at room temperature (24°C ± 2°C), with lighting from 08:00 to 20:00 hours. The animals were cared for in accordance with the principles of the *Guide for Care and Use of Experimental Animals*.

### Induction of Experimental Diabetes

We generated 2 models of experimental diabetes, known as neonatal diabetes, by STZ (Sigma-Aldrich Química S.A., Alcobendas, Madrid, Spain) treatment, as previously described [10, 11]. The n0-STZ model was obtained by a single dose of STZ (100 mg/kg body weight [bw]) dissolved in citrate buffer (0.1 mol/L) at pH 4.5, administered intraperitoneally on the day of birth. The n5-STZ model was induced by a single dose of STZ (80 mg/kg bw) on day 5 after birth. The nondiabetic (ND) control rats received only the citrate buffer, intraperitoneally. At the age of 2.5 months, the body weight and glycemia were measured and an oral glucose tolerance test was performed in all groups. Blood samples were obtained from a cut made at the tip of the animal's tail, and blood glucose was immediately assayed using a glucometer and reactive strips (Glucocard Memory; A. Menarini Diagnostics, San Adrián del Besós, Barcelona, Spain). The rest of the blood was collected into preheparinized Eppendorff tubes, and the plasma was separated by centrifugation and stored at −70°C until immunoreactive insulin (IRI) assays were performed.

### Oral Glucose Tolerance Test

After 18 hours' fasting, all the groups (normal, n0-STZ diabetic, and n5-STZ diabetic rats) were subjected to an oral glucose (0.5 g/kg bw) tolerance test. First a blood sample was taken (time 0), and then glucose was administered via a gastric cannula (0.5 g/kg bw, as a 50% solution in 0.9% NaCl), with the animal awake. Blood samples were taken at the times indicated in Figure 1. Incremental areas under the curves (AUCs) of glucose and insulin were determined by planimetry. Insulin was determined by radioimmunoassay with a rat insulin kit that uses a specifically synthesized antibody against rat insulin (DRG's Instrument GmbH, Marburg, Germany). Only those diabetic animals presenting a clear oral glucose intolerance were selected to form part of the diabetic groups in the study.

### Isolation of Enterocytes and Assay Methods

The disaccharidases (sucrase and maltase) and 6-phosphofructo-1-kinase (PFK-1) activities were determined in enterocytes isolated from the complete small intestine following the method previously described [12]. In brief, the rat was killed by a pentobarbital overdose. The abdomen was cut open, and the small intestine was removed, rinsed with 20 mL of 0.9% NaCl, blotted dry, weighed, and measured under 5-g tension. Enterocytes were then isolated by a method that allows the isolation of metabolically competent enterocytes, based on an EDTA and gentle mechanical treatment of the small intestine mucosa. The disaccharidase activity was determined by the technique of Dahlqvist [13]. PFK-1 activity was measured under maximum-rate conditions by the technique of Kitajima and Uyeda [14]. Protein concentration was determined by the micro-Lowry method (Sigma-Aldrich Química S.A.), using bovine albumin as standard.

The results are expressed as means with their corresponding standard errors of measurement (SEM). The statistical analysis used the Mann-Whitney $U$ test for independent samples.

### RESULTS

Table 1 lists the general characteristics of the experimental animals. In adulthood, the n0-STZ and n5-STZ rats presented hyperglycemia, which was significantly greater in the n5-STZ than in the n0-STZ rats. Insulin values were significantly lower in both diabetes models than in the controls. The body weight was similar between the n0-STZ diabetic rats and the controls, but was lower ($P < .01$) in the n5-STZ group. The weight and length of the small intestine, however, were significantly greater in the 2 diabetes models than in the controls;

**TABLE 1**
General characteristics of nondiabetic (ND) and n0-STZ and n5-STZ diabetic rats measured at age 2.5 months

| | ND | n0-STZ | n5-STZ |
|---|---|---|---|
| Blood glucose (mmol/L) | 5.99 ± 0.07 | $8.77 \pm 0.47^{**}$ | $20.83 \pm 0.63^{**aa}$ |
| Plasma insulin (ng/mL) | 3.77 ± 0.22 | $1.74 \pm 0.53^{*}$ | $1.12 \pm 0.44^{**}$ |
| Body weight (g) | 367 ± 13 | 390 ± 12 | $250 \pm 33^{**aa}$ |
| Small intestine weight (g) | 8.57 ± 0.19 | $10.97 \pm 0.72^{*}$ | $18.25 \pm 0.72^{**aa}$ |
| Small intestine length (cm) | 108 ± 1 | $118 \pm 2^{*}$ | $144 \pm 5^{**aa}$ |

*Note*. Values are the mean ± SEM for 6 animals for each group.
$^{*}P < .05$; $^{**}P < .01$ compared to ND rats; $^{aa}P < .01$ compared to n0-STZ rats.

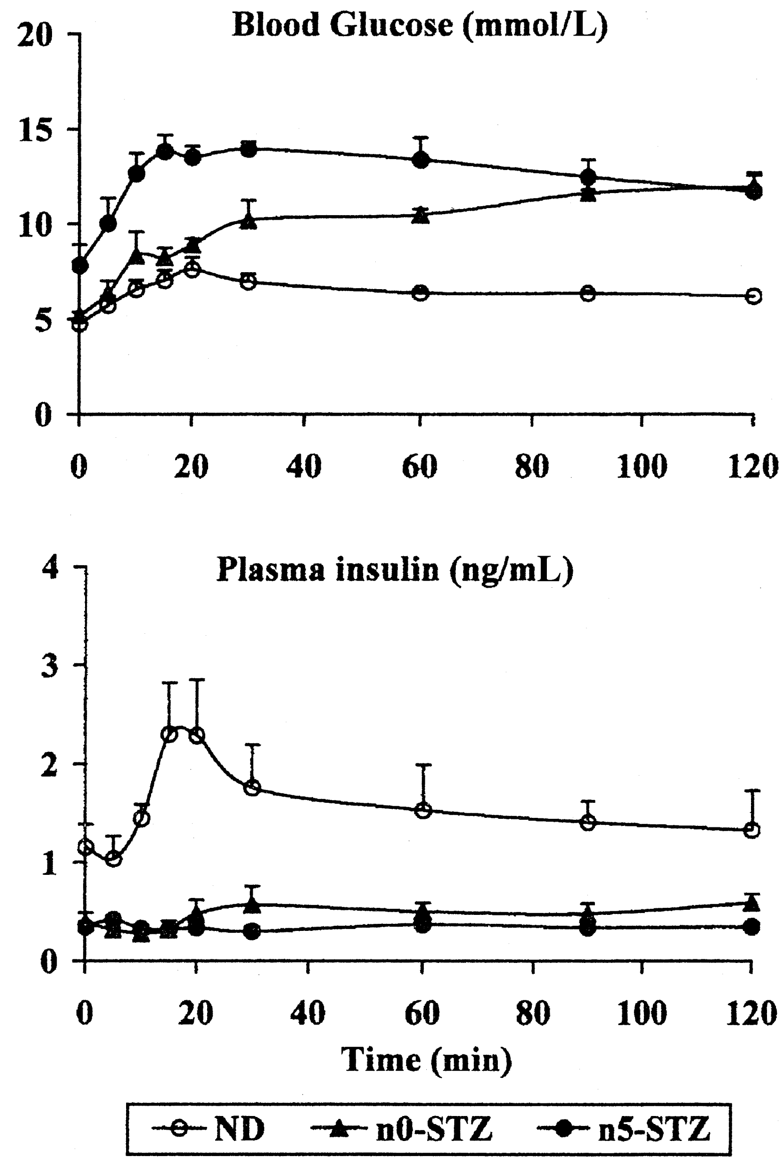

FIGURE 1
Glycemia (mmol/L) and insulinemia (ng/mL) measured during an oral glucose tolerance test after 18 hours of fasting, given to nondiabetic (ND) and n0-STZ and n5-STZ diabetic rats. Values are the mean ± SEM for 6 animals for each group.

and both parameters were significantly greather ($P < .01$) in the n5-STZ versus the n0-STZ rats.

The development of diabetes in the n0-STZ and n5-STZ rats was confirmed by the oral glucose tolerance test (Figure 1). Oral administration of glucose led to a significant increase of the blood glucose AUC (mmol/L · 120 min) in both the n0-STZ model (600 ± 23; $P < .05$) and the n5-STZ model (592 ± 77; $P < .05$) with respect to the ND group (204 ± 16). These changes were accompanied by a lower insulin secretion response (plasma insulin AUC: ng/mL · 120 min) in the 2 diabetes models (n0-STZ: 13 ± 2; n5-STZ: 8 ± 2; $P < .05$) with respect to the ND group (60 ± 14).

As shown in Figure 2, the specific activities of sucrase and maltase in the n0-STZ (89 ± 9 and 266 ± 12 U/g protein) and n5-STZ (142 ± 23 and 451 ± 57 U/g protein) diabetic rats were significantly higher than those in ND rats (66 ± 5 and 228 ± 22 U/g protein). Also, the enterocyte enzymatic activities were higher in the n5-STZ than in the n0-STZ diabetic rats ($P < .05$).

Figure 3 shows the PFK-1 specific activity measured in enterocytes isolated from the small intestine of the 3 groups of rats. The values (mU/mg protein) were significantly lower ($P < .05$) in the diabetic (n0-STZ: 14.89 ± 1.51; n5-STZ: 13.35 ± 3.12) than in the ND (20.54 ± 2.83) rats.

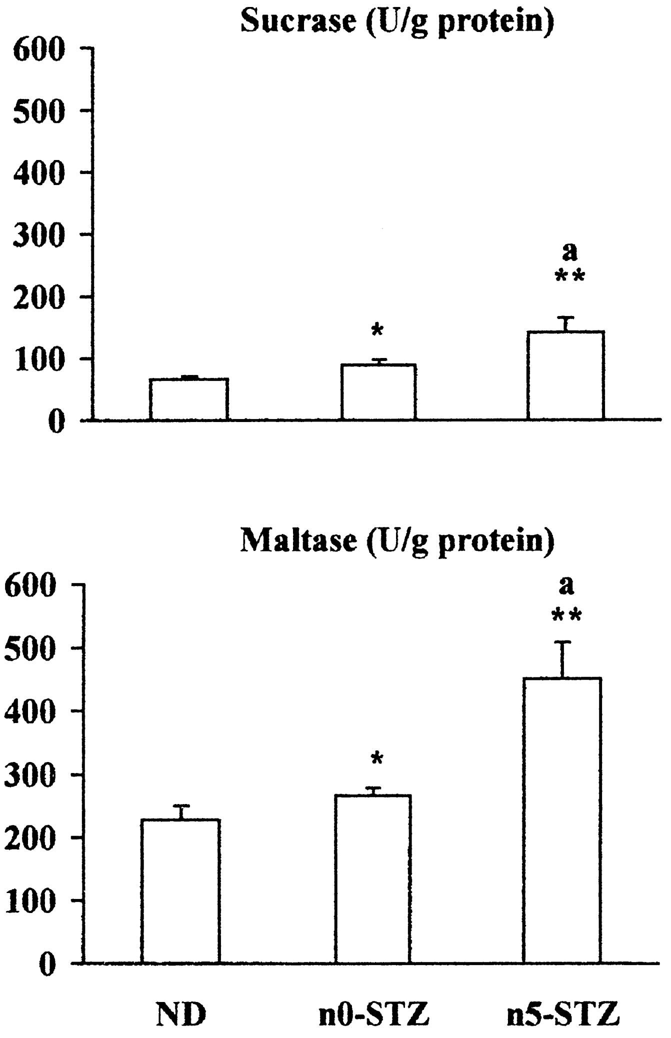

**FIGURE 2**
Sucrase and maltase activities (U/g protein) measured in enterocytes isolated from ND and n0-STZ and n5-STZ diabetic rats. Values are the mean ± SEM for 6 animals for each group. $^*P < .05$ and $^{**}P < .01$ versus ND rats; $^aP < .05$.

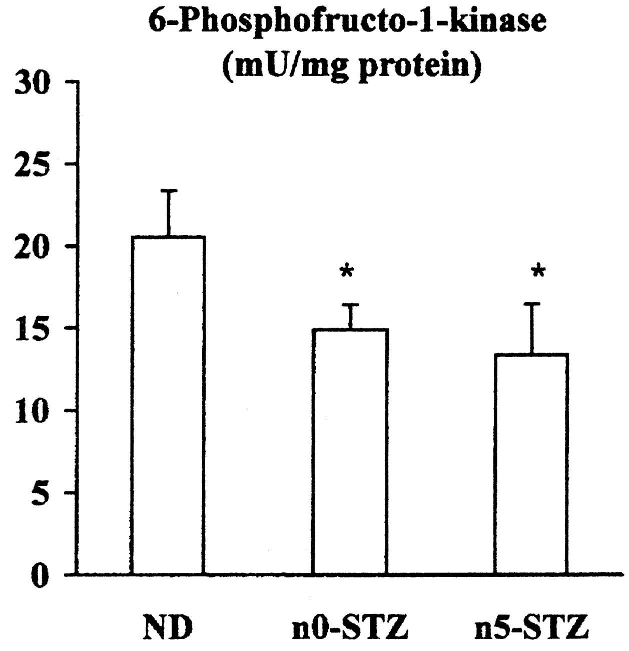

**FIGURE 3**
6-Phosphofructo-1-kinase activity measured in enterocytes isolated from ND and n0-STZ and n5-STZ diabetic rats. Values are the mean ± SEM for 6 animals for each group. $^*P < .05$ versus ND rats.

### DISCUSSION

For the present study, we used 2 models of nonobese type 2–like diabetes, induced by the intraperitoneal STZ administration in the neonatal period (n0-STZ and n5-STZ models). These models had been developed and thoroughly investigated by Portha and coworkers [10, 11, 15, 16]. They showed that defects in insulin secretion and action develop in the n-STZ models, and that these defects in many ways resemble those described in humans. Nevertheless, there have been no studies on how the intestinal function is affected. We found that the length and weight of the small intestine were increased in those 2 models of diabetes. This is in agreement with results that we had previously obtained in n0-STZ diabetic rats [17], and with the results of other models of STZ-induced type 1 diabetes that show hypertrophy of the small intestine mucosa as well as other alterations [18, 19]. The etiology of these morphological alterations is, however, still an issue of debate [20, 21].

It is well known that specific and total activities of the disaccharidases are increased in the mucosa of the small intestine of diabetic animals [4–6, 9] and diabetic patients [3]. This contributes to the increase in the rate of glucose absorption observed both in humans and in animal type 1 diabetes models, and justifies the pharmacological use of intestinal alpha-glucosidase inhibitors in the treatment of diabetes. Our results in diabetic animal models are consistent with the studies that report increased intestinal disaccharidase activities in type 1 diabetic animals. We have also found an increase in sucrase and maltase activities measured in the proximal intestine mucosa scrapings of n0-STZ diabetic rats [17]. Caspary and colleagues [2] describe a reversal or depression of this increase following insulin treatment. Murakami and Ikeda [9] concluded that hyperglycemia is partially responsible for the increased disaccharidase activities in diabetes. Our results support the conclusions of Murakami and Ikeda [9], because the increase in disaccharidase activity was greater in the enterocytes from the n5-STZ diabetic rats, which presented a greater degree of hyperglycemia and lower insulinemia than the enterocytes isolated from the n0-STZ diabetic rats.

Other enzymatic alterations have also been observed in the diabetic intestine. Rats with insulin-deficient diabetes have been reported to have reduced PFK activity in the proximal intestinal mucosa [8] and in enterocytes isolated from the small intestine [7]. We also have found a reduction in PFK-1 activity in the proximal and distal intestinal homogenates of n0-STZ diabetic rats [17]. These results are in agreement with those of the present work with enterocytes isolated from the n0-STZ diabetic rats, where the reduction in PFK-1 activity was 27%, and from the n5-STZ diabetic rats, where the reduction was 35%. In principle, such a reduction in PFK activity could reduce the utilization of glucose by the small intestine in rat models of type 1 diabetes [22, 23], thus contributing to a postprandial hyperglycemia in diabetes.

The effect of diabetes on enzymatic activities and glucose transport has been attributed to hyperglycemia [9], hypoinsulinemia [2], or both. Although the present work was not aimed at studying mechanisms, the results do suggest that the greatest effects are obtained in the most hyperglycemic and most hypoinsulinemic rats.

Our study shows the appearance of enzymatic alterations in enterocytes isolated from 2 models of nonobese type 2 diabetes in rats (n0-STZ and n5-STZ). It also shows that such diabetes produces modifications that favor an increase in glucose absorption rates. The small intestine enzymatic alterations are more pronounced in n5-STZ than in n0-STZ diabetic rats.

### REFERENCES

[1] Mooradian, A. D., Morley, J. E., Levine, A. S., Prigge, W. F., and Gebhard, R. L. (1986) Abnormal intestinal permeability to sugars in diabetes mellitus. *Diabetologia,* **29,** 221–224.

[2] Caspary, W. F., Rhein, A. M., and Creutzeldt, W. (1972) Increase of intestinal brush border hydrolases in mucosa of streptozotocin-diabetic rats. *Diabetologia,* **8,** 412–414.

[3] Tandon, R. K., Srivastava, L. M., and Pandey, S. C. (1975) Increased disaccharidase activity in human diabetics. *Am. J. Clin. Nutr.,* **28,** 621–625.

[4] Caspary, W. F. (1973) Effect of insulin and experimental diabetes mellitus on the digestive-absorptive function of the small intestine. *Digestion,* **9,** 248–263.

[5] Schedl, H. P., Al-Jurf, A. S., and Wilson, H. D. (1983) Elevated intestinal disaccharidase activity in the streptozotocin-diabetic rat is independent of enteral feeding. *Diabetes,* **32,** 265–270.

[6] Díez–Sampedro, A., Milagro, F. I., Berraondo, B., Zulet, M. A., and Martínez, J. A. (1997) Effects of trecadrine, a $\beta$3-adrenergic agonist, on intestinal absorption of D-galactose and disaccharidase activities in three physiopathological models. *J. Pharm. Pharmacol.,* **49,** 873–877.

[7] Madsen, K. L., Ariano, D., and Fedorak, R. N. (1995) Vanadate treatment rapidly improves glucose transport and activates 6-phosphofructo-1-kinase in diabetic rat intestine. *Diabetologia,* **38,** 403–412.

[8] Jamal, A., and Kellet, G. L. (1983) Regulation of mucosal phospho-fructoquinase in the small intestine of the streptozotocin-diabetic rat. *Diabetologia,* **25,** 355–359.

[9] Murakami, I., and Ikeda, T. (1998) Effects of diabetes and hyperglycemia on disaccharidase activities in the rat. *J. Gastroenterol.,* **33,** 1069–1073.

[10] Portha, B., Levancher, C., Picon, L., and Rosselin, G. (1974) Diabetogenic effect of streptozotocin in the rat during the perinatal period. *Diabetes,* **23,** 889–895.

[11] Portha, B., Blondel, O., Serradas, P., Mc Evoy, R., Giroix, M.-H., Kergoat, M., and Bailbe, D. (1989) The rat models of non-insulin-dependent diabetes induced by neonatal streptozotocin. *Diabetes Metab.,* **15,** 61–75.

[12] Watford, M., Lund, P., and Krebs, H. A. (1979) Isolation and metabolic charasteristics of rat and chicken enterocytes. *Biochem. J.*, **178,** 589–596.

[13] Dahlqvist, A. (1964) Method for assay of intestinal disaccharidases. *Anal. Biochem.*, **7,** 18–25.

[14] Kitajima, S., and Uyeda, K. (1983) A binding study of the interaction of beta-D-fructose-2,6,-biphosphate with phosphofructokinase and fructose-1,6-biphosphatase. *J. Biol. Chem.,* **258,** 7352–7357.

[15] Blondel, O., Bailbe, D., and Portha, B. (1989) In vivo insulin resistance in streptozotocin diabetic rats: Evidence for reversal following oral vanadate treatment. *Diabetologia,* **32,** 185–190.

[16] Blondel, O., Bailbe, D., and Portha, B. (1990) Insulin resistance in rats with non-insulin-dependent diabetes induced by neonatal (5 days) streptozotocin: Evidence for reversal following phlorizin treatment. *Metabolism,* **39,** 787–793.

[17] Tormo, M. A., Martínez, I. M., Romero de Tejada, A., Gil-Exojo, I., and Campillo, J. E. (2002) Morphological and enzymatic changes of the small intestine in an n0-STZ diabetic rat model. *Exp. Clin. Endocrinol. Diabetes,* **110,** 119–123.

[18] Mayhew, T. M., and Carson, F. L. (1989) Mechanisms of adaptation in rat small intestine: Regional differences in quantitative morphology during normal growth and experimental hypertrophy. *J. Anat.,* **164,** 189–200.

[19] Mantlee, M., Thakore, E., Atkins, E., Mathison, R., and Davison, J. S. (1989) Effects of streptozotocin-diabetes on rat intestinal mucin and goblet cells. *Gastroenterology,* **97,** 68–75.

[20] Ettarh, R., and Carr, K. (1997) A morphological study of enteric mucosal epithelium in the streptozotocin-diabetic mouse *Life Sci.,* **61,** 1851–1858.

[21] Nowak, T. V., Harrington, B., Weisbruch, J. P., and Kalbfleisch, J. H. (1990) Structural and functional charateristics of muscle from diabetic rodent small intestine. *Am. J. Physiol. (Gastrointest. Liver Physiol. 21),* **258,** G690–G698.

[22] Hanson, P. J., and Parsons, D. S. (1978) Factors affecting the utilisation of ketone bodies and other substrates by rat jejunum: Effects of fasting and diabetes. *J. Physiol.* **278**, 55–67.

[23] Tormo, M. A., Gómez-Zubeldia, M. A., Ropero, F., Muñoz-Casillas, M., Moreno, J. C., and Campillo, J. E. (1995) Experimental streptozotocin-induced diabetes and intestinal glucose metabolism in the rat, in vivo and vitro. *Acta Diabetol.,* **32,** 182–186.

